# Adaptation of gastrointestinal nematode parasites to host genotype: single locus simulation models

**DOI:** 10.1186/1297-9686-45-14

**Published:** 2013-05-28

**Authors:** Kathryn E Kemper, Michael E Goddard, Stephen C Bishop

**Affiliations:** 1 Department of Agriculture and Food Systems, University of Melbourne, Parkville, VIC 3010, Australia; 2 Victorian Department of Environment and Primary Industries, AgriBio, 5 Ring Road, Bundoora, VIC 3083, Australia; 3 The Roslin Institute and the Royal (Dick) School of Veterinary Studies, Easter Bush, Midlothian EH25 9RG, UK

## Abstract

**Background:**

Breeding livestock for improved resistance to disease is an increasingly important selection goal. However, the risk of pathogens adapting to livestock bred for improved disease resistance is difficult to quantify. Here, we explore the possibility of gastrointestinal worms adapting to sheep bred for low faecal worm egg count using computer simulation. Our model assumes sheep and worm genotypes interact at a single locus, such that the effect of an *A* allele in sheep is dependent on worm genotype, and the *B* allele in worms is favourable for parasitizing the *A* allele sheep but may increase mortality on pasture. We describe the requirements for adaptation and test if worm adaptation (1) is slowed by non-genetic features of worm infections and (2) can occur with little observable change in faecal worm egg count.

**Results:**

Adaptation in worms was found to be primarily influenced by overall worm fitness, *viz*. the balance between the advantage of the *B* allele during the parasitic stage in sheep and its disadvantage on pasture. Genetic variation at the interacting locus in worms could be from *de novo* or segregating mutations, but *de novo* mutations are rare and segregating mutations are likely constrained to have (near) neutral effects on worm fitness. Most other aspects of the worm infection we modelled did not affect the outcomes. However, the host-controlled mechanism to reduce faecal worm egg count by lowering worm fecundity reduced the selection pressure on worms to adapt compared to other mechanisms, such as increasing worm mortality. Temporal changes in worm egg count were unreliable for detecting adaptation, despite the steady environment assumed in the simulations.

**Conclusions:**

Adaptation of worms to sheep selected for low faecal worm egg count requires an allele segregating in worms that is favourable in animals with improved resistance but less favourable in other animals. Obtaining alleles with this specific property seems unlikely. With support from experimental data, we conclude that selection for low faecal worm egg count should be stable over a short time frame (e.g. 20 years). We are further exploring model outcomes with multiple loci and comparing outcomes to other control strategies.

## Background

Sheep selected over many generations for low faecal worm egg count (WEC) show large reductions in WEC when infected with gastro-intestinal parasites [1-3]. This reduction in WEC indicates a reduced reproductive capacity, or reduced fitness, for worms in these hosts. A concern often voiced is that worm adaptation to sheep bred for low WEC may occur. The expectation is that natural selection results in worms increasing their fitness (or reproductive capacity) over time and this could erode the benefits of selecting sheep for low WEC. However, to date these expectations have not been realised in experiments [4-7] and, anecdotally, breed differences in WEC appear to be stable across time. In this paper, we investigate whether the expectation of worm adaptation to low WEC sheep is realistic and attempt to determine factors that influence this potential rate of evolution.

A compelling explanation of why worms are not observed to adapt to sheep selected for low WEC is lacking. Numerous authors have put forward possible explanations, including features of the host-parasite relationship that slow the rate of adaptation, such as the skewed distribution of WEC, which results in the majority of worms being harboured by susceptible hosts; and the insensitivity of WEC as a measure of adaptation e.g. [8-10]. However these assertions are rarely (if ever) quantified. It is unclear which factors influence adaptation of worms to hosts that have been selected for improved resistance. More generally, it is important to know which factors might also be applicable to other livestock disease systems (i.e. species and pathogens) such as selection of salmon or chickens for improved resistance to bacterial or viral infections. This investigation aims at identifying the key theoretical parameters that determine adaptation of nematodes in sheep. Results of this study may also give insight into the possible influence of these parameters in other situations.

To explore factors that may affect the rate of parasite adaptation to host genotype, we describe a model that combines interacting descriptions of host genetics, parasite genetics and disease biology. We explore its key parameters and test hypotheses relating to observable rates of parasite adaptation. The model is parameterised using a single locus, gene-for-gene model. We investigate the factors that generate or maintain genetic variation in worms at this interacting locus and test two specific hypotheses relating to worm adaptation i.e. (1) the relative rate of adaptation in worms is slowed by features of the host-parasite relationship, such as the skewed distribution of WEC and the different mechanisms by which sheep reduce WEC, and (2) WEC is an insensitive measure of worm adaptation.

## Methods

### Overview

This paper describes results from two models, referred to as the ‘full’ and ‘reduced’ models. Both models have the same underlying interaction between genotype of the sheep and the worm but the full model also includes non-genetic factors related to the infection of sheep with gastrointestinal worms. Details of the genotype interaction and the worm infection used in the full model are given in the section entitled “Model construction”. Key definitions are that the *A* allele results in ‘resistance’ or low WEC in sheep, compared to the alternate *a* allele, and the *B* allele in worms is favourable for parasitizing sheep with the *A* allele (compared to the alternate *b* allele). In the section entitled “Model interrogation”, we describe five scenarios, which are used to explore the values of the parameters that maintain genetic variation in worms (Scenarios 1 and 2), describe the properties of the worm infection and genotype by genotype interaction (Scenarios 3 and 4) and test the hypotheses (Scenario 5). For simplicity, all simulations aim at maintaining a constant frequency of the *A* allele in sheep flocks over time and therefore we detect ‘adaptation’ of worms to particular flocks by observing an increase in the frequency of the *B* allele in worms. To assess the impact of selecting sheep for lower WEC (i.e. an increase in the frequency of the *A* allele), we simulate a range of sheep flocks with different frequencies for *A* and deduce the impact on the worm population of selecting sheep for lower WEC by interpolation between the different flocks.

### Model construction

#### Interaction between sheep and worm genotypes

The model aims at describing worm adaptation to sheep with low WEC and hence it quantifies only differences between sheep genotypes for which worms possess the genetic potential to adapt. The interaction assumes bi-allelic loci with additive allelic effects (Table 1). The frequency of the *A* allele in sheep is *x* and the frequency of the *B* allele in worms is *p*. Genotype frequencies are given by Hardy-Weinberg proportions [11], such that sheep with *AA* genotype have a population frequency of *x*^*2*^.

**Table 1:** Relative fitness of worm genotypes (per generation) as a function of host genotype

**Worm genotype**		**Sheep genotype**		
		**Susceptible (*aa*)**	**Heterozygous (*Aa*)**	**Resistant (*AA*)**
	frequency	*(1-x)*^*2*^	*2x(1-x)*	*x*^*2*^
Wild-type (*bb*)	*(1-p)*^*2*^	*1*	*1 - ¼s*_*h*_	*1 - ½s*_*h*_
Heterozygous (*Bb*)	*2p(1-p)*	*1 - ½s*_*p*_	*1 - ½s*_*p*_	*1 - ½s*_*p*_
Alternate (*BB*)	*p*^*2*^	*1 - s*_*p*_	*(1+¼s*_*h*_*)(1-s*_*p*_*)*	*(1+½s*_*h*_*)(1-s*_*p*_*)*

*s*_*h*_ = survival advantage of the alternate *B* allele for worms when harboured by sheep carrying the ‘resistance’ *A* allele; *s*_*p*_ = survival trade-off (mortality) for the *B* allele, independent of sheep genotype.

The *B* allele in worms is favourable for parasitizing sheep that carry the *A* allele (i.e. those animals with improved resistance to worms). The magnitude of this survival advantage in worms is determined by the parameter *s*_*h*_ (survival in the host). The model also has capacity for pleiotropic effects for the *B* allele so that the allele may be unfavourable for survival in free-living stages of the worm lifecycle (Table 1) [12]. The degree of pleiotropy is controlled by the free-living survival parameter *s*_*p*_ (survival on pasture), such that the pleiotropic effects do not act when *s*_*p*_ is zero. Therefore, there is the potential for *p* to increase when the worms are harboured by sheep with the *A* allele and for *p* to decrease during the free-living stage. There is a subtle but important distinction between ‘worm fitness’, defined as the success of an individual throughout its lifecycle, and ‘worm survival’, which is relevant to only one aspect of the lifecycle.

#### The reduced model

The reduced model uses only population-level genetic information to predict the expected change in the frequency of the *B* allele in worms (per generation) for a given frequency of the *A* allele in sheep. The generational fitness (Ω) is the average fitness of the worm *bb*, *Bb* and *BB* genotypes, weighted by the expected frequency of the three host genotypes from Table 1. That is:

$$\Omega_{\mathrm{bb}}={\left(1-x\right)}^{2}+2x\left(1-x\right)\left(1-\frac{1}{4}s_{h}\right)+x^{2}\left(1-\frac{1}{2}s_{h}\right)\;$$

$$\Omega_{\mathrm{Bb}}=\left(1-\frac{1}{2}s_{p}\right)$$

$$\Omega_{\mathrm{BB}}=\left(1-s_{p}\right)\left[{\left(1-x\right)}^{2}+2x\left(1-x\right)\left(1+\frac{1}{4}s_{h}\right)+x^{2}\left(1+\frac{1}{2}s_{h}\right)\right]$$

The change in worm allele frequency [11], is then:

(1) $$p'=\left(\frac{p^{2}\Omega_{\mathrm{BB}}+p\left(1-p\right)\Omega_{\mathrm{Bb}}}{p^{2}\Omega_{\mathrm{BB}}+2p\left(1-p\right)\Omega_{\mathrm{Bb}}+{\left(1-p\right)}^{2}\Omega_{\mathrm{bb}}}\right)$$

where *p* and *p'* are the frequency of the *B* allele in the current and next worm generations. In contrast to the full model, the reduced model assumes an infinite population of worms and uses discrete generations to make deterministic predictions of the change in *p*.

#### The full model

The full model includes a flock of sheep grazing on a common pasture. Each sheep has its own level of resistance and worm population, with worms continuously infecting and re-infecting sheep. The worm lifecycle obeys the time-dependent dynamics seen with worm infections and, over a season, animals slowly develop increased immunity to the worm infections. The flock has a skewed distribution of WEC measurements, in accordance with field observations.

The full model is described in full detail in Additional file 1. Briefly, the sheep by worm genotype interaction is expanded into a series of daily steps. Each day, sheep ingest worm larvae and initiate a set of interactions unique to the infected animal’s genotype and the genotype of the larvae ingested on that day. Over time, a proportion of the ingested larvae develop into adult worms. Adult (female) worms produce eggs until they eventually die. The eggs produced by worms on a given day can be observed in each animal via a measurement of WEC. Eggs are deposited on the pasture in the faeces and develop over several days. Initially, newly hatched larvae from these eggs are immature but after 7 days the mature larvae are available for ingestion by the grazing flock. Thus, the model has subpopulations of worms defined by day age-classes, either on pasture or within a host, and a flock of grazing sheep. Each day age-class of worms has its own frequency of the *B* allele (*p*).

We explore three postulated mechanisms for the sheep genotype to influence WEC in sheep: (i) reduce worm establishment, (ii) increase adult worm mortality and (iii) reduce adult egg production [3,13,14]. The *A* allele acts through these mechanisms in sheep, facilitating the potential for worms to adapt in the full model. For each mechanism, the *A* allele reduces WEC by a defined amount *α* (described below), such that homozygous *AA* sheep have a WEC that is *2α* lower than the WEC of homozygous *aa* sheep. In the simulations, each mechanism to reduce WEC is tested independently.

### Model interrogation

#### Genotype by genotype interaction and the maintenance of genetic variation at the interacting locus in worms

Worm alleles at the interacting locus could either be *de novo* mutations or variants that already segregate in the population. The reduced model was used to examine these two situations and the properties of the genotype by genotype interaction. There are two scenarios, as defined in Table 2, which use a range of parameter values for *p*_*0*_ (the initial frequency of the *B* allele in worms), *s*_*h*_ (the survival advantage of worms with the *B* allele in sheep with the *A* allele), *s*_*p*_ (the survival penalty on pasture for the *B* allele) and *x* (the frequency of the *A* allele in sheep). Scenario 1 investigates the impact of changes in *p*_*0*_ on the outcome for *p* over many generations, while other parameters are held constant. This scenario is relevant to study the properties of *de novo* mutations that are initially very rare in the population. Scenario 2 explores the maintenance of segregating variants and the impact of changing the survival advantage (*s*_*h*_) and trade-off (*s*_*p*_) of the *B* allele in worms. The frequency of the *A* allele in sheep was equal to 0.2, 0.5 or 0.8. Interpolation across simulations in Scenario 2 shows the properties of the segregating *B* allele for worms in flocks of sheep with an increasing frequency of the *A* allele.

**Table 2:** Summary of the scenarios tested

**Scenario**	**Number of worm generations or years**^**a**^	***s***_***h ***_**(%)**	***s***_***p ***_**(%)**	***p***_***0***_	***x***	**Model type**^**b**^	**Mechanism to reduce sheep WEC**^**c**^
***Evaluation of the genotype-by-genotype interaction***							
**1**	80 gen	20	0	0.001, 0.01,	1.0	reduced	-
				0.1, 0.3			
**2**	80 gen	0 to 30	0 to 30	0.3	0.2, 0.5, 0.8	reduced	-
***Evaluation of the host-parasite interaction properties***							
**3**	1 yr	-	-	0	0	full	-
**4**	1 yr	25	10	0.5^d^	0.5	full	E, M, F
***Testing hypotheses 1 and 2***							
**5**	20 yr	25	10	0.3	0.0 to 1.0	reduced, full	E, M, F

^a^ the reduced model is defined by worm generations and the full model is defined by years of simulation; results show that there are 2.2 worm generations per year of simulation; *s*_*h*_ = survival advantage of the *B* allele in sheep; *s*_*p*_ = survival trade-off on pasture for the *B* allele; *p*_*0*_ = initial frequency of the *B* allele in worms; *x* = frequency of the low WEC *A* allele in sheep; ^b^ see text for details of the full model, which includes complexities of the host-parasite relationship, and the reduced model, which explicitly excludes these factors; ^c^ postulated mechanisms to reduce WEC in sheep through the ‘resistance’ *A* allele: E = reduced worm establishment, M = increased worm mortality, and F = reduced female worm fecundity; ^d^ to evaluate the effect of the low WEC allele for each mechanism of the sheep ‘resistance’ *A* allele, the worm allele frequency was fixed (*p* = 0.5) during the transition stages of the worm lifecycle.

#### Evaluation of the worm infection under the full model

The properties of the worm infection for the full model were examined in two scenarios under conditions whereby the sheep by worm genotype interaction could not influence the results. These scenarios aimed at demonstrating the properties and appropriateness of the simulated worm infection. Parameters for these scenarios are defined in Table 2 (Scenarios 3 and 4). The aim of Scenario 3 was to evaluate the characteristics of the worm infection in a single season. This scenario used a ‘susceptible’ sheep flock (i.e. *x* = 0) and *p*_*0*_ was zero, therefore there was no potential for the worm population to adapt. Scenario 4 tested the properties of the *A* allele in sheep to ensure that each mechanism to reduce WEC had an equivalent effect. That is, we wanted to calibrate the mechanisms such that homozygous *AA* sheep had a WEC that was *2α* lower than homozygous *aa* (‘susceptible’) sheep. Special conditions were required for this scenario to prevent accumulation of effects throughout a season, in order to ensure equivalent test conditions between the mechanisms at the end of the season, and also because the effect *α* of the *A* allele depends on the frequency of the *B* allele in worms. Effects can accumulate within a season because the genotype profile of the worm population changes continually, affecting subsequent WEC measurements. Thus, the allele frequency of the worm population was fixed to 0.5 during each transition stage (i.e. when ingested as L_3_ larvae in sheep and when first deposited as eggs on pasture). A frequency of 0.5 was chosen to ensure equal numbers of homozygotes for worm genotypes. The average allele effect *α* was calculated as

(2) 121−WECAAWECaa,

where *WEC*_*AA*_ is the average WEC of sheep that are homozygous for the low WEC allele, and *WEC*_*aa*_ is that of homozygous susceptible sheep.

#### Testing hypothesis 1: non-genetic factors in the worm infection cycle can slow the rate of worm adaptation

Hypothesis 1 proposes that features of the host-parasite interaction will slow the rate of adaptation. These factors are either (a) factors involved in worm infection (e.g. a skewed distribution of WEC or overlapping worm generations) or (b) the mechanisms of resistance. This hypothesis was tested by assessing if the reduced model could predict the change in *p* from the full model, using each of the three postulated mechanisms to reduce WEC. Thus, we tested all factors included in the full model that are not involved in the genotype by genotype interaction. Parameters to test the hypothesis are detailed in Scenario 5 (Table 2). The parameters were defined such that worms with the *B* allele can have a 25% survival in sheep that carry the *A* allele but there is a 10% survival trade-off for the *B* allele on pasture (i.e. *s*_*h*_ = 0.25, *s*_*p*_ = 0.1). Sheep with one copy of the *A* allele had approximately a 25% reduction in WEC. Each proposed mechanism by which sheep reduce WEC was tested in turn, for scenarios in which the frequency of the *A* allele was increased incrementally from 0 (susceptible) to 1 (resistant). The initial frequency of the *B* allele in worms (*p*_*0*_) was 0.3. Results presented for these scenarios were the mean of five replicate sheep flocks.

#### Testing hypothesis 2: WEC is an insensitive measure of worm adaptation

Worm adaptation to sheep with the low WEC *A* allele may be expected to result in an increase in WEC over time for these animals, i.e. when worms adapt to sheep with the *A* allele, they increase their egg output and hence WEC. In our model, we expect an increase in *p* (i.e. ‘adaptation’) to be associated with an increase in WEC. We used WEC as an indicator of adaptation for data generated from Scenario 5. A linear model (*y = a + bt*) was fitted to the replicate mean WEC over time. Adaptation to the *A* allele in sheep was declared if the regression coefficient (*b*) was significantly greater than zero (P < 0.001).

## Results

### Model parameters for the sheep-by-worm genotype interaction and the maintenance of genetic variation at the interacting locus in worms

**Figure 1:**
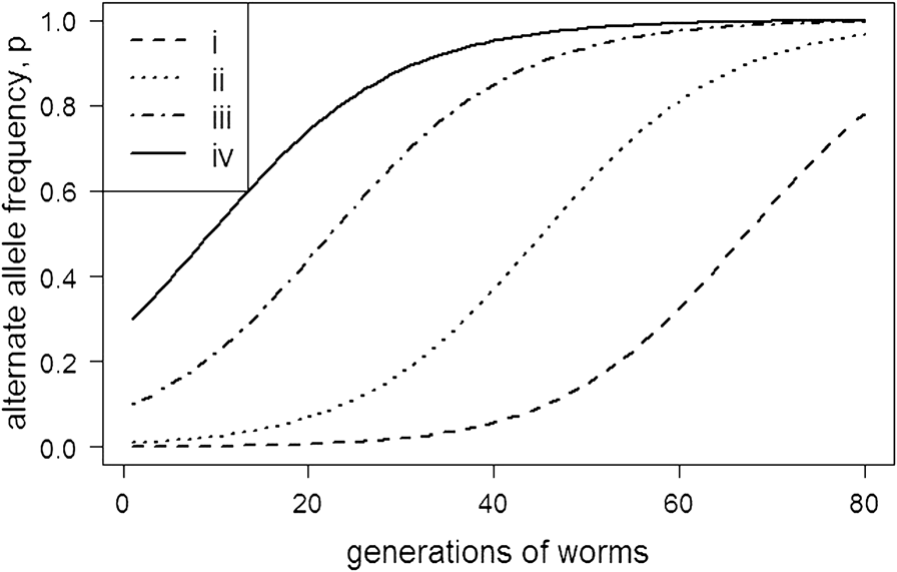
**Changes in the frequency of the alternate *B *allele in worms when the initial frequency of *B *is either (i) 0.001, (ii) 0.01, (iii) 0.1 or (iv) 0.** Under Scenario 1, there is a 20% survival advantage (*s*_*h*_) for the *B* allele in sheep carrying the *A* allele and no mortality trade-off on pasture (*s*_*p*_ = 0).

The reduced model was used to investigate the effect of changes to the underlying parameters for the genotype interaction; *viz*. changes to *p*_*0*_, *s*_*p*_, *s*_*h*_ and *x* (i.e. the initial frequency of the *B* allele in worms, the survival advantage and trade-off for the *B* allele and the frequency of the “resistance” *A* allele in sheep). The results from Scenario 1 are important when considering *de novo* mutations as a source of genetic variation for adaptation in worm populations. This is because *de novo* mutations are expected to be very rare alleles (i.e. a very low *p*_*0*_). The results indicate that changing *p*_*0*_ had little impact on the final outcome for the *B* allele but that *p*_*0*_ did affect the number of generations required before an observable change in *p* occurred (Figure 1). Thus, worms adapt (*p* tends to 1) when the *B* allele increases worm survival in sheep with the *A* allele and when the *B* allele has no pleiotropic effects, regardless of the initial frequency of the *B* allele. Changing *p*_*0*_ affected the total number of generations required until fixation but had no affect on the rate of change at a given allele frequency. For example, an allele that was initially very rare (*p*_*0*_ = 0.001) took 80 generations to achieve fixation compared to an allele that was initially at a higher frequency (i.e. *p*_*0*_ = 0.1). Thus, *de novo* mutations are expected to behave like other alleles with similar properties (i.e. *s*_*h*_ and *s*_*p*_) but observable changes for these alleles will be slower compared to similar alleles at higher frequencies.

**Figure 2:**
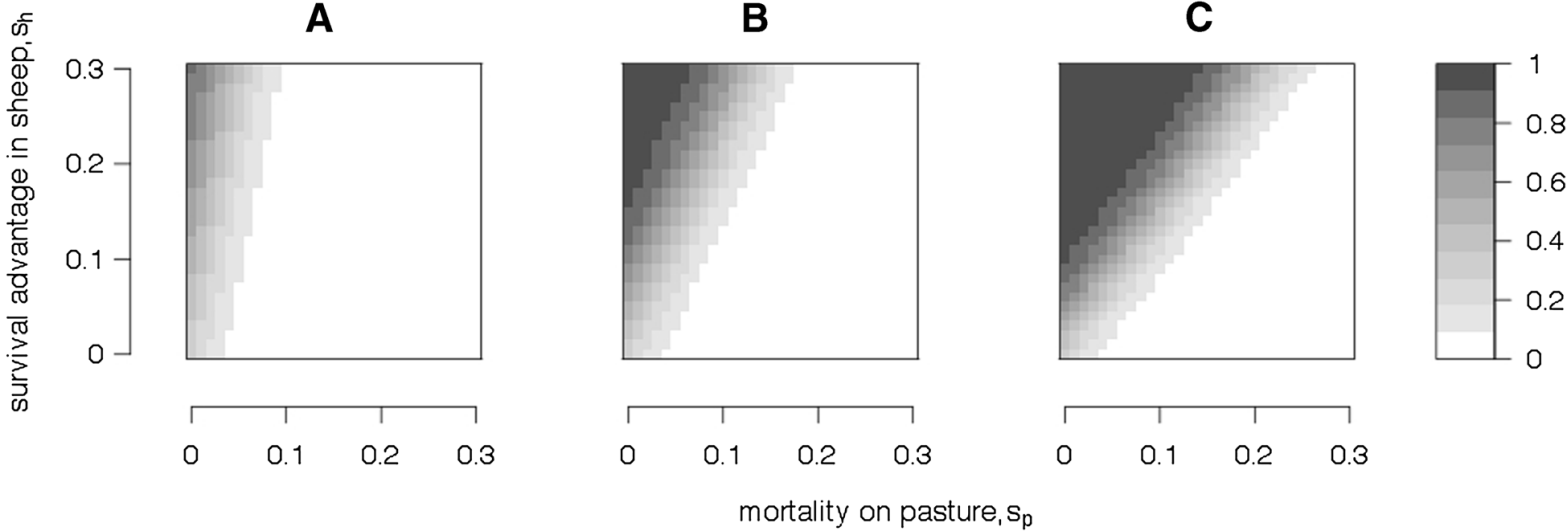
**Frequency of the alternate *B *allele in worms after 80 generations when the interacting *A *allele in sheep is (A) 0.2, (B) 0.5 or (C) 0.8.** Under Scenario 2, the *B* allele survival advantage in sheep carrying the *A* allele (*s*_*h*_, y-axis) and pasture mortality (*s*_*p*_, x-axis) range from 0 to 30%.

Scenario 2 investigated changing the magnitude of the survival effects (*s*_*h*_ and *s*_*p*_) of the *B* allele. Changes in these parameters had dramatic impacts on the final outcome for the *B* allele. Figure 2 summarises results from many simulations using the reduced model, when the *A* allele in sheep had a frequency of 0.2, 0.5 or 0.8. The results indicate that for most of the tested parameter combinations, worms adapt either by fixing (*p* = 1) or completely losing (*p* = 0) the *B* allele. If the survival trade-off of the *B* allele on pasture is greater than its survival advantage in sheep, then the *B* allele will be lost. Since *x* ≤ 1, worm survival on pasture is always more important than worm survival in the host and thus in most cases when *s*_*p*_*≥ s*_*h*_, then *p* tends to 0. This is because the *B* allele is advantageous to worms only when they are harboured by sheep with the low WEC *A* allele (which is uncommon if the frequency of *A* is low), while the survival trade-off for the *B* allele acts on all larvae while on pasture. Thus, the relative importance of survival on pasture depends on the frequency of the *A* allele in sheep. For example, a mutation in worms with a 30% survival advantage in sheep (*s*_*h*_ = 0.3) and a 15% survival trade-off on pasture (*s*_*p*_ = 0.15) would be lost when the frequency of the *A* allele in sheep was low (*x* = 0.2), but would remain segregating if the frequency of the *A* allele was higher (i.e. *x* = 0.5).

If the mutations available for adaptation come from segregating variants at the interacting locus in worms, they must persist after many generations of natural selection. Results from Figure 2 show the type of variants that segregated in the worm population after 80 generations of natural selection. These results indicate a grey area of segregating variants where the *B* allele in worms is neither fixed nor lost. However, given (e.g.) 200 or 1000 generations, this grey area of segregating alleles would be even smaller. For polymorphism to be maintained indefinitely, the homozygous *BB* and *bb* worm genotypes are required to have equal fitness (i.e. *p'* = *p* from equation (1) only when sp=1−ΩbbΩBB). Thus over many generations, only alleles where the survival advantage is precisely balanced by the survival trade-off remain segregating. Some unbalanced alleles may also appear to segregate (or drift) because as sp+ΩbbΩBB tends to 1, the rate of change in the frequency of the *B* allele tends to 0. If adaptation in worms occurs by a change in frequency of segregating variants, then these alleles must affect worm survival but must also have a neutral or near neutral overall effect on worm fitness. Otherwise natural selection would act to fix the favourable allele in worms regardless of the worm resistance exhibited by the sheep. The values for the survival advantage and the trade-off for alleles which segregate indefinitely depend on the frequency of the low WEC *A* allele in sheep. As the frequency of the *A* allele increases, the relative importance of survival on pasture decreases (i.e. the polymorphic combinations in Figure 2 rotate clockwise from vertical, *s*_*p*_ ≈ 0, to 45˚ from vertical, *s*_*p*_ tends to *s*_*h*_, as the frequency of *A* increases). Interestingly, the number of parameter sets for which alleles remain segregating (i.e. the size of the grey area) does not depend on the frequency of the *A* allele. For example, the grey area is an equal size when only one (*x* = 1.0) or all three (e.g. *x* = 0.2) sheep genotypes are present, implying that heterogeneity in sheep does not increase worm heterogeneity in our model.

The *B* allele at the interacting locus only remains segregating after many generations when there is no overall fitness effect for the allele; either the survival advantage of the *B* allele in sheep with the *A* allele is balanced by the survival trade-off on pasture, or the *B* allele does not affect worm survival. However, this balance depends on the frequency of the *A* allele. Thus, if we take a given sheep population and imagine selecting for an increase in frequency of the *A* allele (i.e. increased resistance to worms or a reduced WEC), then worm alleles that previously had no fitness effect may become favourable and begin to increase in frequency. The rate of increase in the *B* allele is determined by the magnitude of change in the frequency of the *A* allele, i.e. the change in the frequency of the *A* allele determines how unbalanced the survival advantage and trade-off in worms becomes. If the frequency of the *A* allele is slightly increased, then there is only a small change in the selection pressures that act on the worms (i.e. a small rotation in the grey area) and, over time, the frequency of the *B* allele increases slowly. If there is a large increase in the frequency of the *A* allele, then the change in the selection pressures for the worm population is greater and this will cause a more rapid increase in the frequency of the *B* allele. In summary, if adaptation in worms is to be observed from segregating variants, the mutation needs to change from having a neutral to having a favourable effect on worm fitness. The magnitude of this change in fitness determines the rate of adaptation, i.e. the rate of increase in frequency of the favourable *B* allele.

#### Properties of the worm infection from the full model

**Figure 3:**
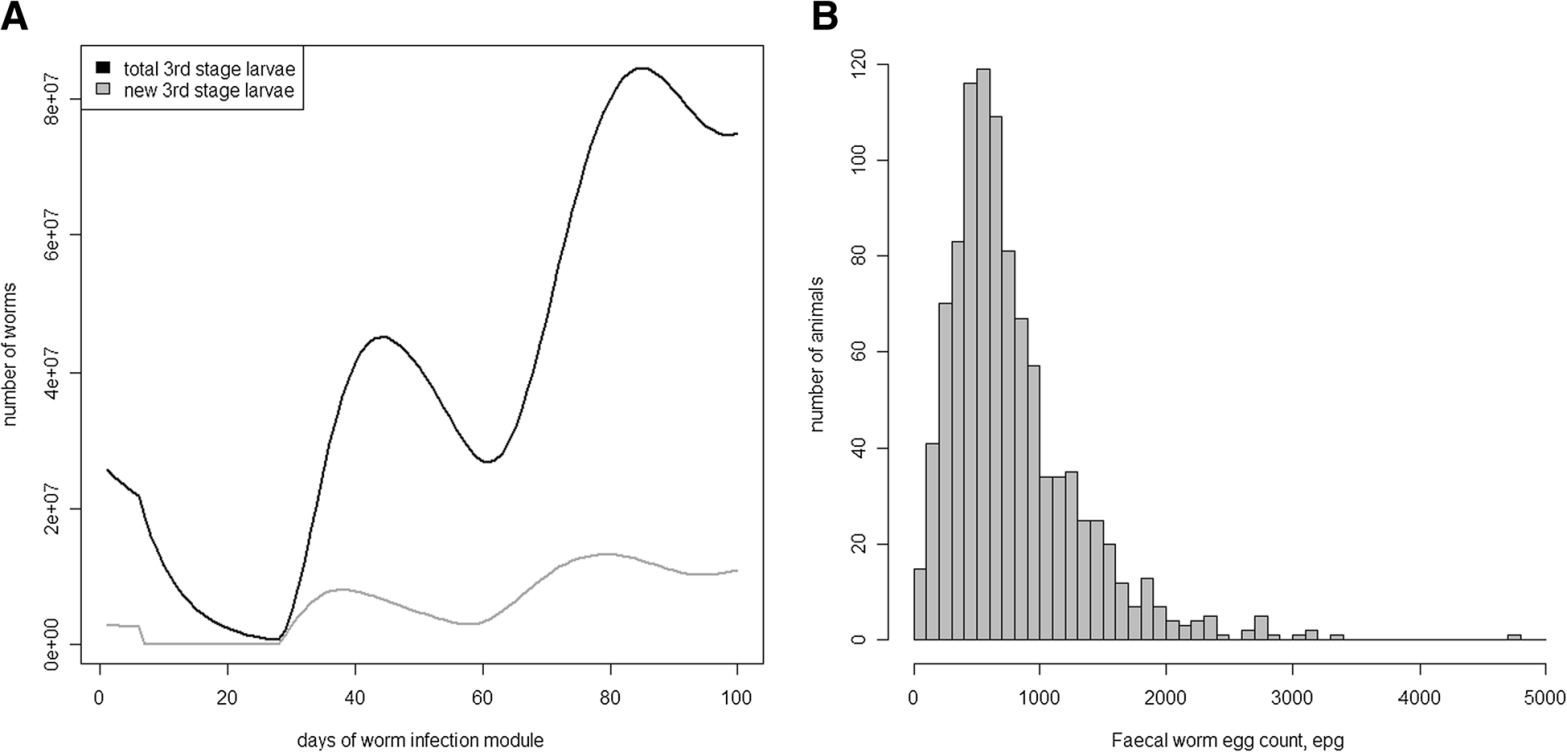
**Total and new L**_**3 **_**larvae on pasture (A) and the distribution of faecal worm egg count on day 100 (B).** Results are from a single replicate of a susceptible sheep flock under Scenario 3.

Scenario 3 aims at investigating the epidemiological properties of the full model without the complication of genetic variation in either the sheep or worm populations. The degree of pasture contamination shows the presence of a pasture reservoir of worm larvae (Figure 3). There are two annual peaks in total pasture contamination, the first at around day 45 and the second at day 85; and two slightly earlier peaks in new infective L_3_ stage larvae on pasture. These peaks are caused by two successive generations of worms infecting the sheep. WEC and worm burden (not shown) show skewed distributions, which is characteristic of these traits. Mean adult burden was 19 310 and mean WEC 769 eggs per gram (epg). The degree of skewness in WEC agrees with previous experimental results [7]. The average generation length for the worm population was 45 days. Hence one year (or 100 days of simulation) is equivalent to 2.2 worm generations, in accordance with field observations [15,16]. These results indicate that our model adequately captured the key epidemiological characteristics of worm infections that we aimed to include in our model.

The effect of the low WEC *A* allele in sheep and the corresponding change in the frequency of the *B* allele in worms is reported in Table 3, taking a census at day 100. For the purpose of testing the model, we held the frequency of the *B* allele for ingested larvae and new eggs on pasture at 0.5, preventing accumulation of changes to worm allele frequency within a season. This procedure was only used when testing the model to ensure correct parameterisation. Depending on the mechanism implemented, sheep with one copy of the *A* allele either reduce the proportion of larvae that establish by 0.029 (untransformed scale), increase daily mortality by 0.038, or reduce female fecundity by 16 eggs per worm. The low WEC allele (*A*) reduced WEC at day 100 by approximately 25% (or 100–150 epg) for all mechanisms; hence the WEC of homozygous *AA* sheep was 40 to 60% of the WEC of homozygous *aa* sheep. Table 3 demonstrates some important consequences of the mechanisms to reduce WEC and of the epidemiological model. These include:

**Table 3:** **Effect of the low WEC *A *allele in sheep depending on the mechanism by which sheep reduce WEC**

**Mechanism of the *A *allele**	**Sheep genotype**	**Mean adult worms**^**a**^			**WEC mean**^**b**^		**Effect of allele *A *(α%)**	**Mean pasture L**_**3**_^**c**^		
		**Count**	***p***	**Age (days)**	**Count (epg)**	***p***		**Max nb (x10**^**7**^**)**	***p***	**Age (days)**
**E**	*aa*	11 769	0.500	31.3	487	0.500				
	*Aa*	8 776	0.516	31.2	352	0.516	22.4	4.72	0.489	15.2
	*AA*	6 610	0.531	31.2	268	0.531				
**M**	*aa*	12 581	0.500	30.7	501	0.500				
	*Aa*	8 916	0.518	27.3	356	0.518	29.7	4.93	0.488	15.2
	*AA*	5 067	0.534	25.6	203	0.534				
**F**	*aa*	11 134	0.500	31.3	449	0.500				
	*Aa*	11 344	0.500	31.3	354	0.508	21.9	4.22	0.488	15.2
	*AA*	11 267	0.500	31.3	252	0.516				

Postulated mechanisms to reduce WEC in sheep through the ‘resistance’ *A* allele: E = reduced worm establishment, M = increased worm mortality and F = reduced female worm fecundity; epg = eggs per gram; ^a^number, age, and frequency of the alternate *B* allele (*p*) for adult worms; ^b^average effect on WEC (*α*) and *p* for deposited eggs; ^c^number, frequency of *p* and age for infective L_3_ larvae on pasture.

1. A reduction in the number of adult worms as *x* increases (from *aa* to *Aa* to *AA* sheep), when the *A* allele reduces worm establishment or increases adult mortality. The adult worms are younger when harboured by sheep with an *A* allele that acts by increasing adult worm mortality. As expected, there is no change in the number of adult worms in sheep with the *A* allele when it acts to reduce worm fecundity.

2. There is an increase in the frequency of the *B* allele for adult worms harboured by sheep with the *A* allele when the mechanism of the *A* allele is to reduce worm establishment or increase adult worm mortality. Similar to point 1 above, no differences in the frequency of the *B* allele are evident between *AA*, *Aa* and *aa* sheep genotypes when the *A* allele acts by reducing worm fecundity.

3. The frequency of the *B* allele is unchanged from adult worms to eggs when the *A* allele acts by either reducing worm establishment or increasing adult mortality. However, the frequency of the *B* allele is greater in the eggs than in adult worms when the *A* allele acts by reducing worm fecundity.

4. The frequency of the *B* allele increases in sheep (i.e. in sheep with the *A* allele) but decreases during the pasture stage of the lifecycle. That is, there is a survival trade-off on pasture for the *B* allele.

5. The magnitude of increase in the frequency of the *B* allele differs between the three mechanisms of the *A* allele. When the *A* allele acts by reducing adult worm fecundity, the change in allele frequency is approximately half of that when the *A* allele acts by reducing establishment or increasing adult worm mortality. This is insightful for the fecundity mechanism. When the *A* allele acts by reducing worm fecundity, it affects only the female worms that contribute to the next generation and not the male worms (i.e. the effect is sex-limited). Thus, the magnitude of increase for *p* in the eggs is only half of that when the mechanism of the *A* allele acts on both sexes.

#### Testing hypothesis 1: non-genetic features of the host-parasite relationship slow the rate of worm adaptation

**Figure 4:**
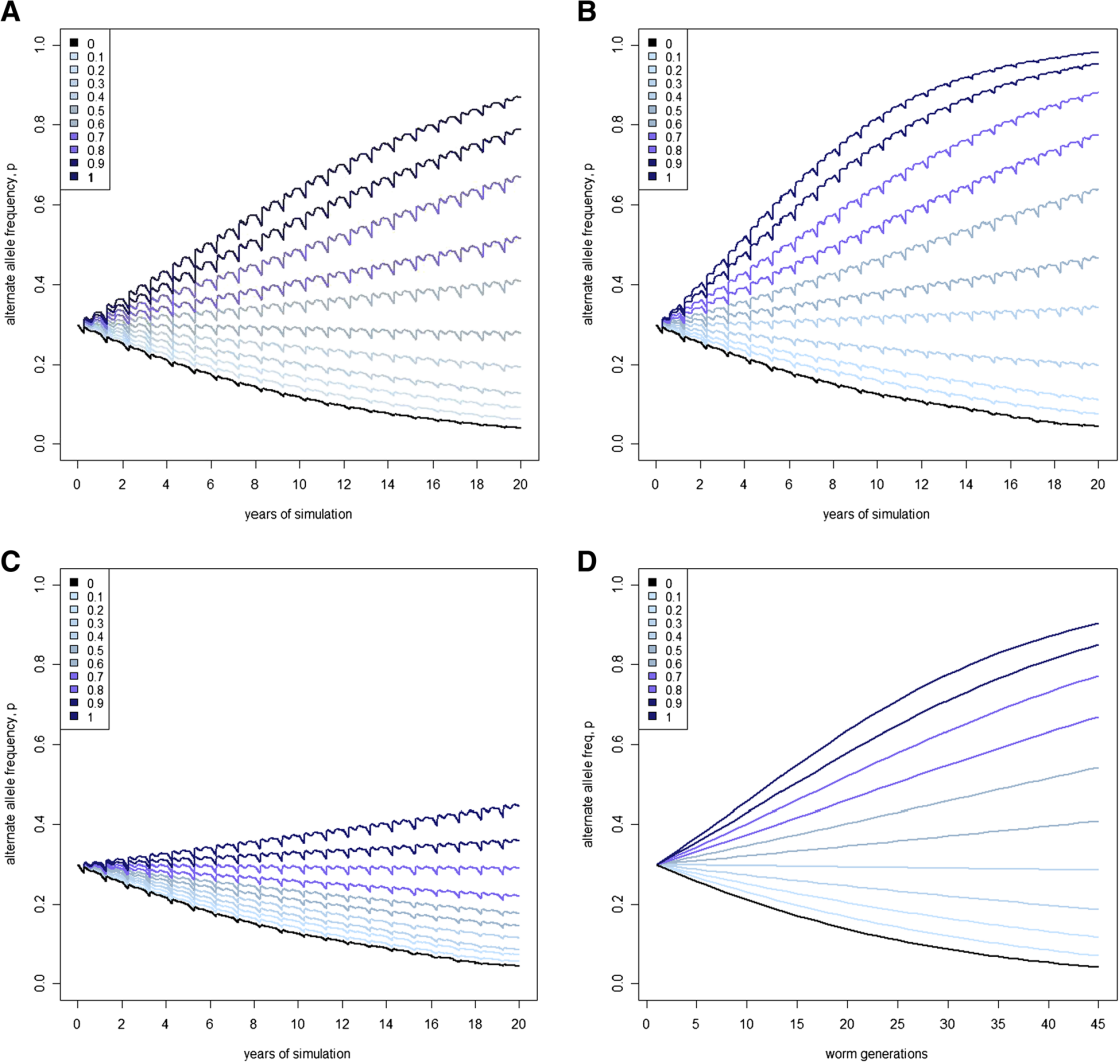
**Mean frequency of the *B *allele in worms during 20 years of simulation using either the full (A-C) or reduced (D) model.** Under scenario 5, there is a 25% survival advantage (*s*_*h*_) and 10% pasture mortality (*s*_*p*_) for the *B* worm allele. The mechanisms by which sheep reduce faecal worm egg count (WEC) are (**A**) reduced worm establishment, (**B**) increased worm mortality, or (**C**) reduced worm fecundity; the frequency of the low WEC *A* allele in sheep is investigated at 11 levels, from low (light blue) to high (dark blue) frequencies and also when the allele is absent (black).

The change in frequency of the worm *B* allele is shown over 20 generations for Scenario 5 in Figure 4 (plots A, B, C). For most frequencies of the *A* allele in sheep, the *B* allele in worms either moves towards fixation or loss. The rate of change (i.e. the slope of the line) depends on the frequency of the *A* allele in sheep. Thus, as the sheep population has lower WEC (i.e. as *x* increases), the slope describing the change in the frequency of the *B* allele in worms is increasingly positive, indicating that the *B* allele is favourable. Adaptation was most rapid when the low WEC *A* allele in sheep was fixed (*x* = 1.0).

Comparing the change in allele frequency in worms between the outputs of the four models allows the first hypothesis to be tested (Figure 4). If non-genetic factors involved with the worm lifecycle, such as the skewness of WEC and a reservoir of infective larvae on pasture, had an effect on the rate of worm adaptation, then there would be observable differences between outputs from the full and the reduced models. Second, if differences in the rate of adaptation are due to the mechanisms by which WEC is reduced in sheep, there would be differences between the three sets of results from the full model. Figure 4 shows a strong similarity between the allele frequency change for the establishment and mortality mechanisms from the full model and the reduced model, bearing in mind that one year approximates 2.2 parasite generations. There may be a slightly higher final frequency of the *B* allele for worms that parasitize sheep with the mortality mechanism, compared to results from the establishment mechanism or from the reduced model. This is probably due to the non-linearity of the mortality mechanism and the accumulation of effects over time due to changes in the age structure of the worm population. In contrast, the results from the reduced model do not predict the results from the full model when the mechanism to reduce WEC is by decreasing adult worm fecundity. For this mechanism, the rate of change in the frequency of the *B* allele in worms was negative at most frequencies of the *A* allele in sheep. Hence the *B* allele is mostly unfavourable for the worms. This arises because selection pressure is reduced for this mechanism, as observed in Table 3. The results from Figure 4 suggest that most features of the host-parasite interaction that were considered in the full model, including the between-host distribution of worms, have little impact on the rate of adaptation in worms. However, when sheep reduce WEC by reducing worm fecundity, a slower rate of adaptation in worms is predicted.

Notably, although the rate of change in the frequency of the *B* allele in worms is lower when WEC is reduced through the fecundity mechanism compared to other mechanisms, the rate of loss for the *B* allele in the susceptible population (*x* = 0) is similar for all four sets of results. This property is best explained with reference to the gene-by-gene interaction defined in Table 1. If only susceptible *aa* sheep are considered, then worm fitness is only determined by the value of *s*_*p*_. This parameter is constant for this scenario regardless of the mechanism and hence Figure 4 shows the same rate of loss for all unselected sheep. The sex-limited action of the fecundity mechanism only affects the relative fitness of the worm genotypes when *x* is greater than zero. In effect, the sex-limited action halves the values of *s*_*h*_ in Table 1. This changes the balance for fitness between worm genotypes, leading to results for the fecundity mechanism being different from results for the other mechanisms.

#### Testing hypothesis 2: WEC is an insensitive measure of worm adaptation

**Figure 5:**
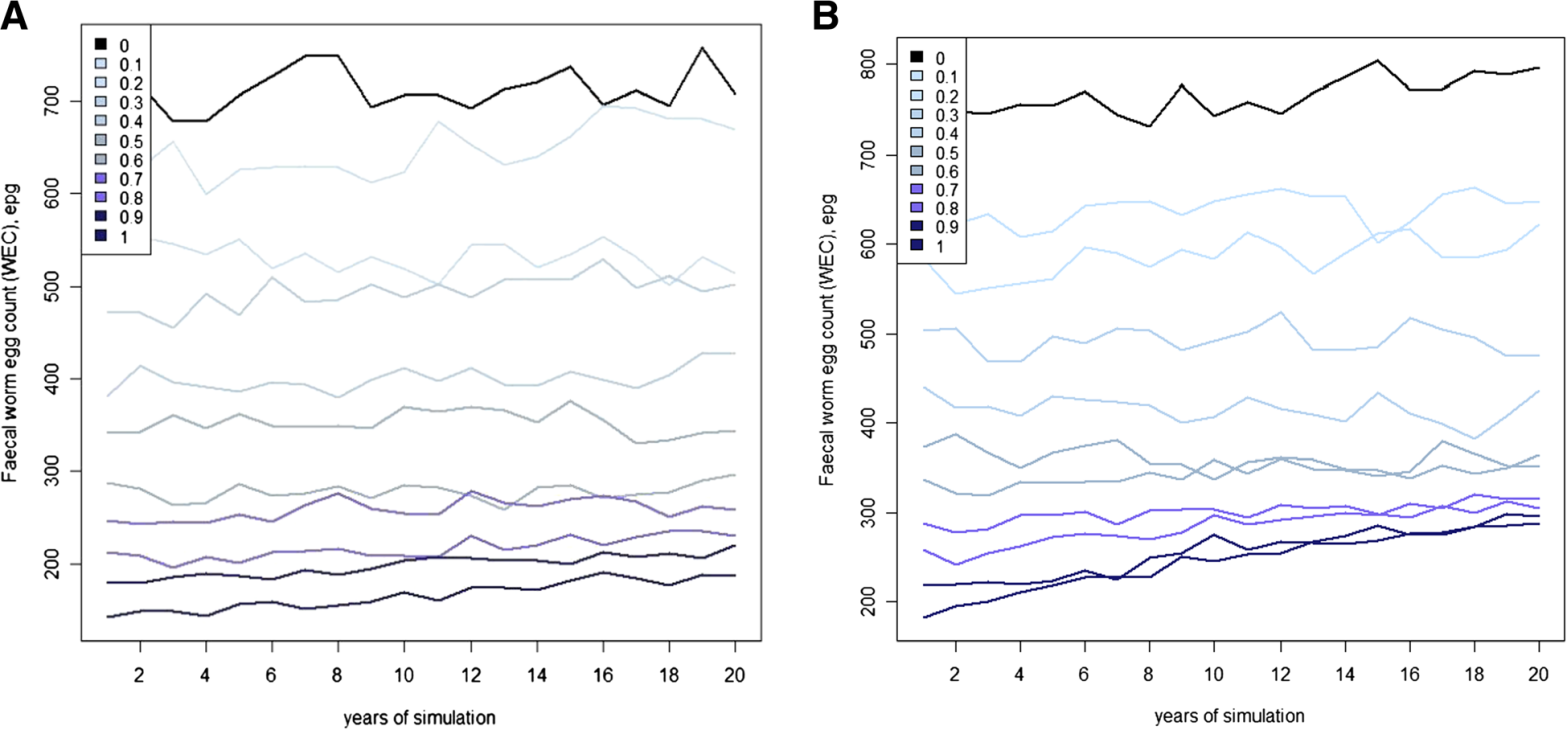
**Mean faecal worm egg count (WEC) during 20 years of simulation of worm infection in sheep under Scenario 5.** Mechanism by which sheep reduce WEC are (**A**) reduced worm establishment or (**B**) increased worm mortality; the frequency of the low WEC *A* allele in sheep was investigated at 11 levels, from low (light blue) to high (dark blue) frequencies and also when the allele is absent (black).

The flock average WEC measurements showed the clear effect of a reduction in WEC with increasing frequency of the *A* allele in the sheep population for each of the resistance mechanisms (Figure 5 and Table 4). However, the WEC for sheep flocks in which the *A* allele was fixed (i.e. *x* = 1.0) was much lower than that estimated by the effect of the resistant allele. That is, the homozygous *AA* genotype sheep had a WEC of 40% to 60% of the *aa* sheep in Table 3 but the WEC of the *AA* flock was 20 to 30% of the *aa* flock (using the average of years 1 to 5) in Scenario 5. This difference is primarily driven by the within-year effects on pasture contamination. For example, the ‘susceptible’ sheep flocks from Table 4 have an initial WEC of ~700 epg, while the *aa* animals from Table 3 have a WEC of ~475 epg. The susceptible flock from Scenario 5 has a higher WEC because pasture contamination for the flock is higher (i.e. there are no sheep with the *AA* genotype, while in Scenario 4 all three genotypes contribute to pasture contamination) and the mortality on pasture for worms is lower because the frequency of the *B* allele in worms is lower (i.e. *p*_*0*_ is 0.3 in Scenario 5 compared to 0.5 in Scenario 4). In Scenario 5, an additional ~ 25% reduction in WEC is observed in an *AA* flock (*x* = 1) because of reduced worm burden and larval intake arising from lower larval contamination from low WEC sheep and increased mortality for *B* allele worms on pasture.

**Table 4:** The regression of replicate mean WEC on time for flocks in Scenario 5

**Mechanism of the *A *allele**	**Frequency of the *A *allele (*x)***	**Flock average WEC (epg)**		**Regression coefficient (epg/yr)**		**∆*p***
		**Year 1**	**Year 20**	**Estimate**	**s.e.**	
E	0.0	710	710	0.72	0.87	
E	0.1	640	670	**3.21**	**0.80**	**Decrease**
E	0.2	540	510	−1.07	0.58	
E	0.3	470	500	**2.08**	**0.50**	**Decrease**
E	0.4	380	430	1.16	0.45	
E	0.5	340	340	−0.17	0.50	
E	0.6	290	300	0.39	0.36	
E	0.7	250	260	1.02	0.37	
E	0.8	210	230	**1.62**	**0.26**	**Increase**
E	0.9	180	220	**1.83**	**0.18**	**Increase**
E	1.0	140	190	**2.54**	**0.20**	**Increase**
M	0.0	720	800	**2.94**	**0.56**	**Decrease**
M	0.1	640	650	1.22	0.65	
M	0.2	580	620	2.39	0.66	
M	0.3	500	480	−0.08	0.62	
M	0.4	440	440	−0.89	0.54	
M	0.5	370	370	−1.40	0.43	
M	0.6	340	350	**1.81**	**0.39**	**Increase**
M	0.7	290	310	**1.07**	**0.27**	**Increase**
M	0.8	260	320	**3.62**	**0.24**	**Increase**
M	0.9	220	300	**4.35**	**0.30**	**Increase**
M	1.0	180	290	**5.53**	**0.29**	**Increase**
F	0.0	720	800	**2.94**	**0.56**	**Decrease**
F	0.1	660	660	0.10	0.80	
F	0.2	570	540	−1.13	0.58	
F	0.3	480	520	**4.02**	**0.63**	**Decrease**
F	0.4	410	430	−0.52	0.53	
F	0.5	360	340	0.08	0.53	
F	0.6	310	320	−0.69	0.47	
F	0.7	250	240	−0.33	0.45	
F	0.8	230	210	0.55	0.37	
F	0.9	180	170	−0.30	0.29	
F	1.0	140	170	**0.99**	**0.16**	**Increase**

Postulated mechanisms to reduce WEC in sheep through the ‘resistance’ *A* allele: E = reduced worm establishment, M = increased worm mortality and F = reduced female worm fecundity; epg = eggs per gram; *s*_*h*_ = 25%; *s*_*p*_ = 10%; bold characters = coefficients significantly different to zero (P < 0.001) with the directional change in frequency for the alternate *B* allele in worms (∆*p*).

Notably, the WEC in flocks for which obvious adaptation occurred (e.g. the *x* = 1.0 flock from Figures 4B and 5B) did not recover to the level seen in *aa* sheep flocks (*x* = 0). This is because the *B* worm allele suffers a survival penalty on pasture in our simulations, lowering the number of infective larvae on pasture when the worm population has an increased frequency of the *B* allele. Thus, although worms improve survival by adapting to sheep with *AA* genotype, overall fitness for worms with the *B* allele remains lower than wild-type worms in susceptible sheep because of the allele’s pleiotropic effects.

Changes in WEC over time were generally a poor indicator of adaptation because of its high annual fluctuations (Figure 5). The annual fluctuations are not due to variation in environmental factors such as climate (these parameters were constant) but are likely caused by random processes in the model, such as daily variation in feed intake. Although the significant regression of WEC on time indicated quantifiable adaptation, reflecting the increase in the frequency of the *B* allele (Table 4), the changes in WEC were rather small and noisy. For example, significant adaptation was indicated when the *A* allele reduced establishment for the *x* = 0.1 and 0.3 flocks but not for the intermediate *x* = 0.2 flock. As above, the increase in WEC was sometimes associated with a loss of the *B* allele because of its pleiotropic effects on pasture survival. It is unlikely that this magnitude of change in WEC would be observable under field conditions, where the level of larval challenge varies across years, and changes are further masked by climatic fluctuations.

## Discussion

This paper examined the conditions required for adaptation of worms at a single locus to sheep with an allele that confers low WEC, and tested two hypotheses related to rates of worm adaptation. For the former, our model was set up with specific conditions to facilitate adaptation of worms to host genotype by including interactions between sheep and worm genotypes (i.e. Table 1). This is necessary because when an allele in the worm consistently has favourable (or unfavourable) effects and no interaction with the genotype of the sheep, it would always be under positive (or negative) selection and, thus, such an allele could not be responsible for adaptation to specific sheep genotypes (i.e. low WEC sheep). Hence, we used a gene-for-gene model where the genotypes of sheep and worms could interact. The second requirement for adaptation is that worms need genetic variation at this locus. This variation could be through either *de novo* or segregating variants. However, *de novo* mutations are, by definition, initially rare alleles and we found that they may require many generations in the worm population before an appreciable change in allele frequency is observable. Furthermore, these alleles are quickly subjected to natural selection and purged if unfavourable. We found that the mutations that remained segregating after several generations of exposure to sheep had very specific values for their survival advantage and trade-off. Only mutations for which the survival advantage in sheep was precisely balanced by their detrimental pleiotropic effects remained segregating in the long term. Our model assumed the detrimental effects comprised reduced survival on pasture but, equally, these effects could have occurred elsewhere in the lifecycle of the worm. Thus the basic requirements for adaptation in our model were presence of loci in the host and worm that interact and genetic variation in both the worm and host locus.

The presence of genetic variation suitable for adaptation of worms to sheep with improved resistance is unknown. A high degree of molecular polymorphism is observed in worm populations [17,18] and this variation could be equated with adaptive potential. Our results, particularly from Scenario 2, suggest that polymorphisms in worms with appreciable frequencies (and therefore ‘old’ mutations segregating in the population) are likely neutral, or near neutral, for overall worm fitness. This is because worms have infected sheep for many generations and natural selection will have had opportunity to maximise worm fitness. Polymorphisms that segregate in worm populations could affect fitness if they were younger, and thus exposed to fewer generations of natural selection, or if, for example, they have transient effects on fitness [19]. However, the persistence of these polymorphisms will be inversely related to their net effect on fitness because any allele with strong beneficial effects will be rapidly fixed. Our model explored antagonistic pleiotropy as a mechanism to maintain polymorphism in worms and, although experimental evidence for antagonistic pleiotropy at individual loci is scarce [20], Jørgensen [12] reports reduced survival (larval development) for worms at the population level in a line of sheep selected for low WEC. The presence of polymorphisms in worms with antagonistic effects on survival is unknown but our model demonstrates that such a mechanism may be required to maintain polymorphism at a locus with properties suitable for adaptation of worms to sheep genotype. Without pleiotropy, natural selection rapidly purges unfavourable alleles from the worm population and genetic variation is lost at the locus. We expect that most of the observed molecular polymorphisms in worms will have small effects on worm fitness but recognise that segregation of alleles with antagonistic pleiotropy or of young mutations with near-neutral effects on fitness remain a possibility.

The final requirement for worm adaptation to sheep genotype is selection pressure. That is, there must be a difference in survival between worm genotypes that depends on sheep genotype. In our model, selection pressure depended on the survival advantage and trade-off for the allele at the interacting locus in the worm (allele *B*) and the allele frequency at the interacting locus in sheep (allele *A*). Our model behaved in predictable and consistent ways across all scenarios with respect to worm adaptation (i.e. the change in the frequency of the interacting worm allele, *p*). Results from Scenario 5 showed that, for a specific mutation, flocks with extreme frequencies of the interacting allele in the host (*x* = 0 or *x* = 1 for allele *A*) led to the most rapid rate of adaptation. This was because, under the conditions of Scenario 5, the *B* allele remained segregating in the worm population after several generations when *x ~* 0.5 and frequencies of either *x* = 0 or *x* = 1 caused the most disruption in this balance. The requirements for an interacting locus, i.e. genetic variation and selection pressure, should also apply to other host-pathogen systems.

Given the ‘ideal’ starting conditions for worms to adapt, we were able to test two specific hypotheses with our model. The first hypothesis tested if the (non-genetic) features of the host-parasite interaction slowed the observed rate of adaptation. Some features, including a skewed distribution of WEC in sheep, overlapping generations of worms and a reservoir of worm larvae on pasture, primarily impacted the generation interval of worms. Thus, after calibrating the outputs by generation interval in the worm, a simpler reduced model was able to accurately predict the results from the full model that included key host-parasite interactions. The reduced model showed that the outcome for the *B* allele in worms is essentially determined by the selection pressure per generation, i.e. the selection differential for each sheep genotype and the proportion of worms exposed to each sheep genotype, and by the generation length for the worm. Accurate estimates of these two parameters are important to apply these findings to other host-pathogen systems, although in practice this is not a trivial exercise.

An important outcome of the model was that limiting female fecundity as the mechanism by which selection of sheep reduces WEC was found to halve the selection pressure on the worm population compared to other mechanisms for WEC reduction. This was because of the sex-limited action of this mechanism, whereby a female worm that is able to overcome the *A* allele in sheep has its favourable genotype diluted by mating with unselected male worms. Experimental evidence also suggests an important role for reduced worm fecundity in low WEC sheep [3,21-24], where WEC in sheep with immature immune responses seems to be suppressed by reduced worm fecundity rather than a (partial) reduction in total number of worms. Other host-parasite systems also suggest slower adaptation when hosts reduce pathogen reproduction (or infection rate) as compared to pathogen numbers. For example, modelling in malaria shows imperfect vaccines that limit pathogen growth (i.e. numbers) are more likely to cause an evolutionary response in the pathogen compared to an infection-blocking mode of action [25]. Quantitative support for this prediction is evident in the successive adaptation of the Marek's disease virus to repeated releases of vaccines that limit tumor growth rather than viral reproduction or propagation [26,27]. Here, we make a similar favourable conclusion about mechanisms that limit pathogen reproduction, as compared to pathogen numbers, but our results are obtained through the sex-limited action of the fecundity mechanism, in contrast to other studies, which exploited within-host competition between pathogen strains as mechanism for adaptation.

The second hypothesis investigated whether WEC could be used to detect adaptation in worms. We found that WEC over time was very noisy, even in the idealised situation of constant environmental conditions. There was evidence for adaptation by an increase in WEC over time, but in some parameter sets worms adapted by losing the favourable *B* allele, i.e. by reducing their ability to parasitize sheep carrying the *A* allele but improving their ability to survive on pasture. Thus, an increase in WEC over time did not necessarily imply that worms had improved their ability to parasitize sheep. The observed increase in WEC was also smaller than naively expected, i.e. WEC did not increase to the same level as in susceptible (*x* = 0) sheep, even when worms completely fixed the favourable *B* allele. This occurred because the antagonistic pleiotropy of the worm *B* allele. Thus, although worms improved their ability to parasitise sheep by increasing the frequency of the *B* allele, they also reduced their survival on pasture, therefore lowering larval intake, worm burden and WEC of sheep.

Our conclusions of course depend on the assumptions used in our model, i.e. the properties of the interacting loci in sheep and worms (Table 1), which represent our understanding of the processes involved in adaptation. For the source of genetic variation in worms, we make a distinction between segregating and *de novo* mutations, where the former has implicit restrictions on the effects of polymorphisms present in worm populations because of natural selection. We expect *de novo* mutations to add genetic variation each generation and, given the large effective population size of worms, this may be a substantial contribution. For example, Barton [28] argued that it is possible to observe a specific nucleotide substitution in each generation of the worm population, assuming a population of 10^6^ individuals (i.e. a mutation rate of 10^-8^/3, where 10^-8^ is the mutation rate per nucleotide and there are possible three nucleotide substitutions). However the forces that act on *de novo* mutations are the same as we have modelled for segregating mutations, except that the allele frequency for *de novo* mutations is very low. Hence the selection differential for the worm population in each sheep genotype and the proportion of the worm population exposed to this selection pressure will determine if a new mutation persists, and the rate at which it will increase in frequency should it persist. However to facilitate differential adaptation (i.e. adaptation specifically to sheep with *A* genotype), these new mutations still require them to have favourable effects on worm fitness and increase in frequency in low WEC sheep but be unfavourable (or neutral) in sheep that are not selected.

To complete our findings, several issues need to be further studied. The first limitation of our model is that we consider only a single interacting locus. Fisher’s infinitesimal model (a very large number of loci each with a negligible effect) and recent experimental evidence suggests that WEC in sheep is a highly polygenic trait [29]. It might be expected that presence of multiple loci affecting WEC would reduce the selection pressure acting on each worm locus, although this is yet to be rigorously explored. Secondly, a suitable method to test for adaptation, in theory and practice, might be to measure WEC in a cross-factorial design with artificial infection e.g. [7]. This approach should limit the impact of antagonistic effects on the detection of adaptation because each sheep would be exposed to the same larval intake, but it does negate effects that accumulate during the season. Finally, the ability of worms to adapt to anthelmintics should be comparable to adaptation under the framework we have established. It is well known that worms can adapt to anthelmintics e.g. [30] and at first sight this outcome differs from results presented here. However the selection pressures placed on worms by anthelmintic administration are much greater than those exerted by host genetic effects. These factors can be accounted for through the available parameters and are the subject of ongoing research.

## Conclusions

Our model suggests that detectable adaptation of worms to sheep that are selected for low WEC is unlikely in the short term. This is because mutations in the worm that have properties suitable for adaptation, i.e. mutations that are favourable for survival in low WEC sheep but unfavourable in unselected sheep, may take many generations to increase in frequency or are likely to be neutral (or near neutral) with respect to overall worm fitness in the current population. Even when we assumed ideal conditions and adaptation was observed in our model, the expected increase in WEC as a result of adaptation was small relative to the temporal fluctuations in the trait and also small relative to the reduction in WEC expressed by the sheep. The selection pressure applied to the worm population was a key factor for worm adaptation, with the selection differential between worm genotypes and the proportion of the worm population exposed to the selection pressure (i.e. the frequency of the low WEC allele in sheep) being key parameters.

## Competing interests

The authors declare that they have no competing interests.

## Authors’ contributions

KEK contributed to the model design, carried out the model implementation and wrote the first draft of the paper; MEG and SCB contributed to the model design. All authors have read and approved the manuscript.

## Supplementary Material

Additional file 1 **Details of the host-parasite interaction model.** This file contains all equations and parameters to reconstruct the full host-parasite interaction model. Click here for file
