# Supplementary material for: Adaptation of gastrointestinal nematode parasites to host genotype: single locus simulation models
Source: Genet Sel Evol. 2013 May 28;45(1):14. doi: 10.1186/1297-9686-45-14 (PMC3704967; doi:10.1186/1297-9686-45-14)
Supplement: Additional file 1 — Details of the host-parasite interaction model. This file contains all equations and parameters to reconstruct the full host-parasite interaction model. [file 1297-9686-45-14-S1.doc]

## Details of the host-parasite interaction model

## The full host-parasite interaction model consists of two modules, the animal module and the worm infection module. Each module completes a cycle once per year of simulation. The animal module generates sheep phenotypes at each of the proposed mechanisms to control worm infections and a genotype for the resistance mechanism according to the input parameters (e.g. *x*, the frequency of the *A* allele in sheep). The worm infection module takes individuals from the animal module and infects them with worms from pasture. The input variables for the worm infection module include *s_h_* (survival advantage of the alternate allele in sheep), *s_p_* (survival trade-off on pasture for the alternate allele) and the mechanism of the *A* allele to reduce WEC in sheep. The two modules are described in detail below.

**Animal module**

**Sheep phenotypes**

The animal module produces phenotypes for each of the proposed mechanisms to control worm infections and a genotype for each sheep. Each sheep has a phenotype for larval establishment, adult mortality and female fecundity; these phenotypes are the sum of genetic and environmental effects . Phenotypes change over time during the worm infection module (see below). The phenotypes for the *j^th^* sheep (at time zero) for establishment (*E_j,t_*), mortality (*M_j,t_*) and fecundity (*F_j,t_*) are:

E_j,0_ = exp (E_G,j_ + E_E,j_)

M_j,0_ = M_G,j_ + M_E,j_

F_j,0_ = F_G,j_ + F_E,j_

Thus, the phenotypes are the sum of genetic (*E_G,j_ M_G,j_ F_G,j_*) and environmental (*E_E,j_ M_E,j_ F_E,j_*) deviations. Note that the phenotype for larval establishment has an exponential transformation to create the characteristic skewed distribution of worms among sheep.

The genetic component for each phenotype in sheep is the sum of allelic and polygenic effects. The polygenic component describes the background genetic effects to which the worm population cannot adapt (i.e. a base or unselected population). Alleles may or may not be segregating for a particular trait, depending on the mechanism under investigation. Each sheep inherits alleles from its parents at random, where γ is the number of *A* alleles (i.e. γ = 0, 1 or 2 for *aa, Aa* or *AA* genotypes). The genetic component of each trait *T* (i.e. *M_G,j_*, *E_G,j_*, *F_G,j_*,) is calculated as:

$T_{G,j}=\frac{1}{2}T_{poly,sire}+\frac{1}{2}T_{poly,dam}+T_{MS}+\gamma\alpha'$ [A1]

i.e. the sum of mid-parent average of polygenic effects, the Mendelian sampling term [*T_MS_*, distributed N(0*,*½*σ^2^_poly_*)] and the effect of the *A* allele on the trait (*α'*). Environmental deviations for all traits sampled from N[0, (*σ^2^_poly_/h^2^_poly_*)(*1 - h^2^_poly_*)], where *h^2^_poly_* is assumed to be 0.30 for all traits.

**Random mating scheme**

The parents of the first cohort of animals at the beginning of each scenario are generated according to the input parameters. Thereafter, sires and dams are selected at random. Progeny are split into male and female lambs (50:50), with 20 % of males and 50 % of females retained for breeding. Male progeny are used immediately as ram lambs and are mated to the mature ewe population. Young females are maintained as juveniles for one year and used in the subsequent mating to replace 25 % of the mature ewe population.

**Worm infection module**

**Overview**

The worm infection module takes input from the animal module as sheep phenotypes (*E_j.0_, M_j,0_, F_j,0_*) and genotypes for the low WEC allele (i.e. *γ*). The module completes daily cycles and calculates the number of worms in each day age-class and their associated worm allele frequency (Figure A1). Each worm infection starts with the same initial pasture contamination and thus the environment (compared to field conditions) is relatively stable across time. The frequency of the alternate *B* allele for worms on pasture in a new year is determined by the average allele frequency from pasture from the previous infection cycle. The model is described below, starting with daily changes to the worm numbers (i.e. death by old age and egg production) and then daily changes to *p*. Importantly, worm death and egg production is dependent on *p* in the age-class and, when in the parasitic stages, on the genotype of the sheep. This differential survival of worms in sheep genotypes enables the interaction described in Table 1 (main text) to operate. A full list of symbols used in the following section is found in Table A1.

Eggs produced

Immature ageing [A2]

Mature ageing [A3]

Larvae ingested

Larval development [A6]

Ageing infective larvae [A7]

Probability of larvae

reaching maturity [A9]

Probability of reaching infective larvae [A5]

*Worm infection module, updated daily*

**PARASITIC STAGE**

**FREE-LIVING STAGE**

Egg production [A4]

Larvae eaten [A8]

A5

WEC [A19]

day 100

Figure A1 The worm infection module consists of worms in day age-classes in either parasitic or free-living life stages.

Shown are a single sheep (for the parasitic stage) and the free-living stage; numbers in parentheses refer to equations (see text) which determine the number of worms moving between age-classes or transitioning between life stages; worm allele frequencies are also updated daily; faecal worm egg count (WEC) is determined on day 100.

Table A1 List of variables, parameters and constants used in the full epidemiology model

| **Variable** | | **Description** |
| --- | --- | --- |
| *B_i,j,t_* | | worm burden; number of worms in age-class *i* in sheep *j* at time *t* |
| *C_i,t_* | | pasture contamination; number of worms in age-class *i* at time *t* |
| *O_i,j,t_* | | egg output; number of eggs produced by worms in age-class *i* in sheep *j* at time . |
| *E_j,t_, M_j,t_, F_j,t_* | | host phenotype for sheep *j* at time *t*; for daily worm establishment (*E_j,t_*), worm mortality (*M_j,t_*) and female worm fecundity (*F_j,t_*).; host phenotypes have underlying genetic and environmental effects |
| *L_j,t_* | | larval intake; for sheep *j* at time *t* |
| *FI_j,t_* | | feed intake; for sheep *j* at time *t* |
| *p _i,(j),t_* | | frequency of the *B* allele in worms for age-class *i*, in sheep *j* (in the parasitic stage) at time *t* |
| *W_E,i.j.γ_, W_M,i.j.γ_, W_F,i.j.γ_, W_C,i,j_* | | the relative establishment (*W_E,i,j,γ_*), survival in sheep (*W_M,i,j,γ_*), survival on pasture (*W_C,i,j_*) or fecundity of worms (*W_F,i,j,γ_*) for age-class *i* of worms in sheep *j* (with genotype *γ*); these variables enable the sheep by worm genotype interaction through the daily establishment, survival and reproduction parameters (*ω_AA.γ_* *ω_Aa.γ_* & *ω_aa.γ_* ) |
|  | |  |
| *ω_AA.γ_, ω_Aa,γ_, ω_aa,γ_* | | the daily effect of the worm genotype, in a sheep with *γ* copies of the resistance allele (in parasitic stage); the daily effects are equivalent (per generation) to *s_h_* and *s_p_* from Table 1 (main text); when effects act on an age-class of worms over successive days the value of *ω* is *s_h_* or *s_p_* to the inverse power of the average number of days for which the term is applied to the age-class |
| *x* | | frequency of the low WEC allele *A* in the sheep population |
|  | |  |
| **Constant** | **Value** | **Description** |
| *J* | 1 000 | total number of sheep per worm infection cycle |
| *I* | 100 | oldest age-class of worms |
| *γ* | 0, 1, 2 | number of copies of the *A* allele in sheep |
| *ρ* | 0.05 | daily mortality rate of L_3_ larvae on pasture |
| *ε* | 0.045 | probability of developing to an L_3_ larvae |
| *η* | 20 | available herbage per animal (kg) |
| *β* | 50 000 | daily fecundity burden threshold |
| *δ* | 200 | undigested material (g/kg) |
| *q* | 0.005 | immune constant, establishment |
| *m* | 200 | immune constant, mortality |
| *f* | 90 | immune constant, fecundity |

**Changes to worm numbers during parasitic aging**

Each day, worms move from the *i* to the *i+1* age-class and the number of worms present in each age cohort (*B_i,j,t_*) reduces due to mortality. Immature (< 21 days old, *B_1:20,j,t_*) and mature (*B_21..I,j,t_*) worms (where *I* is the oldest age-class of worms) worms have different ageing functions. This is because larval establishment is defined as survival from ingestion to a mature worm (see below). Thus ageing in the parasitic phase occurs according to:

$B_{i+1,j,t+1}=B_{i,j,t} W_{M,\gamma}$ *i* = 1..20 [A2]

$B_{i+1,j,t+1}=B_{i,j,t} W_{M,\gamma}\left(1-M_{j,t}\right)$ *i* = 21...*I* [A3]

where *B_i,j,t_* is the number of worms in the age-class *i* for individual *j* on day *t*, *W_M,γ_* is the worm survival parameter for sheep with *γ* copies of the *A* allele, *M_j,t_* is an individual’s daily adult mortality phenotype at time *t*.

**Transition between parasitic and free-living stages**

Egg production and deposition onto pasture represent the transition from parasitic to the free-living stages. Worms have a prepatent period of 20-days and egg production commences with the 21^st^ age-class. This is the only stage in the lifecycle during which the total number of worms has the potential to increase. For each sheep, egg production in age-class *i* of mature worms is determined by

$O_{i,j,t}=\frac{1}{2}B_{i,j,t}W_{F,\gamma}F_{j,t}$ i = 21…*I* [A4]

where 50% of mature worms are assumed to be female, *W_F,γ_* is worm reproduction parameter for sheep with *γ* copies of the low WEC allele *A* and *F_j,t_* is a sheep's daily worm fecundity at time *t*. Total eggs produced for sheep *j* is then the sum of all eggs produced for all age-class (i.e. *∑O_21:I,j,t_*).

The number of new larvae on pasture is the sum of all eggs produced by all animals (where J is the total number of animals on pasture). The number of new larvae on pasture is also corrected for losses during the non-infective stages (*ε*, the probability a deposited egg develops to a 3^rd^ stage larva). Thus, at time *t* the new pasture contamination is:

$C_{1,t}=\varepsilon\sum_{j=1}^{J} \sum_{i=21}^{I} O_{i,j,t}$ [A5]

**Changes to the number of worms on pasture**

Daily losses of worms on pasture depend on the age of larvae. Losses independent of worm genotype in the non-infective stages (< 7 days) are accounted for by *ε* and losses independent of worm genotype during infective stages are determined by *ρ*. The worm survival parameter (*W_C_*) depends on worm genotype and acts daily on all worms on pasture. Infective larvae are removed from pasture if eaten by sheep. Thus, ageing for non-infective (*C_1:6,t_*) and infective larvae (*C_7:I,t_*) occurs according to:

$C_{i+1,t+1}=C_{i,t}W_{C}$ *i* = 1..6 [A6]

$C_{i+1,t+1}=C_{i,t}W_{C}\left(1-\rho\right)\left(1-\frac{\sum L_{1:J,t}}{\sum C_{7:I,t}}\right)$ *i* = 7...I [A7]

where *C_i,t_* is the number of worms in age-class *i* at time *t*, and the proportion of larvae remaining after the previous day is represented by $\left(1-\frac{\sum L_{1:J,t}}{\sum C_{7:I,t}}\right)$ where *∑L_1:J,t_* is the previous day’s total larval intake for all animals and *∑C_7:I,t_* = previous day’s total infective larvae.

**Transition between free-living and parasitic stages**

The ingestion of larvae from pasture and their establishment in the host is the worm's transition from free-living to parasitic forms. Larval intake per sheep varies randomly according to feed intake [*FI_j,t_* ~ N(2, 0.1); kg where *FI_j,t_* > 0] and depends on the number of available larvae on pasture (i.e. *∑C_7:I,t_*), the total number of animals (*J*) and available herbage per animal (*η*). Hence, the larvae ingested for sheep *j* at time t (*L_j,t_*) is given by:

$L_{j,t}=\frac{\Sigma C_{7:I,t}FI_{j,t}}{J \eta}$ [A8]

Ingested larvae establish in the host on the day following ingestion with probability *E_j,t_*. The new worm burden is calculated as:

$B_{1,j,t}=L_{j,t}E_{j,t}W_{E,\gamma}$ [A9]

where *W_E,γ_* is the worm establishment parameter dependent on sheep genotype (γ).

**Calculating the worm survival and reproduction parameter**

Survival on pasture, establishment of larvae, adult worm mortality and reproduction are determined by the frequency of the alternate allele in worms, and the worm genotypes interact with the sheep genotype (i.e. Table 1). The worm survival and reproduction parameters (*W_C_, W_E,γ_, W_M,γ_, W_F,γ_*) mediate this interaction. The terms have a more complex form than those in Table 1 because of the differences between daily (i.e. *ω_AA,γ_*) and generational parameters (i.e. *s_h_*). For the establishment of larvae and female fecundity, the relationship is linear and the same as *s_h_* from Table 1, i.e. *ω_aa,2_* = 1 - ½*s_h_* or *ω_Aa,1_* = 1. However, the effects accumulate over time for adult worm mortality and survival on pasture, hence there is a non-linear relationship with *s_h_* (i.e. the number of worms dying each day depends on the number present which, in turn, depends on number of worms present on the previous day, and so on). The relationship is (for example), where *z* is the average age of the worm in the host or on pasture. This daily effect is an approximation of the effect each generation because the minimum number of worms in an age-class is 1. Thus, *s_h_* and *s_p_* can be approximated in the full model by multiplying the daily survival parameter (*ω*) by the average number of times it is applied to a particular age class of worms.

*W_C_, W_E,γ_, W_M,γ_, W_F,γ_* are the sum of the daily survival or reproduction parameters for each worm genotype (e.g. *ω_AA,γ_*), weighted by the expected frequency of that genotype. When the allele frequency for the alternate allele in worm age-class *i* in sheep *j* at time *t* is *p_i,j,t_* (thus, *q_i,j,t_* is the frequency of the wild-type allele and *p_i,j,t_* + *q_i,j,t_* = 1), survival in the host is

$W_{M,i.j,\gamma} =p_{i,j,t}^{2} \omega_{AA,\gamma} + 2p_{i,j,t}q_{i,j,t} \omega_{Aa,\gamma} + q_{i,j,t}^{2}\omega_{aa,\gamma}$ [A10]

where the survival of the worm genotypes (*ω_AA_,_γ_ ω_Aa_,_γ_, ω_aa_,_γ_*) is dependent on *γ*. The mechanisms to reduce WEC in sheep are tested independently, and so if (for example) *W_M,γ_* ≠ 1, then *W_E,γ_* and *W_F,γ_* = 1.

**Changes to worm allele frequencies during ageing**

The survival parameters (*W_B,γ_* and *W_C_*) change the allele frequency in the worm population as worms grow older. The new allele frequency is given by:

$p_{i+1,j,t+1}=\left(\frac{p_{i,j,t}^{2}\omega_{AA,\gamma}+p_{i,j,t}q_{i,j,t}\omega_{Aa,\gamma}}{p_{i,j,t}^{2}\omega_{AA,\gamma}+2p_{i,j,t}q_{i,j,t}\omega_{Aa,\gamma}+q_{i,j,t}^{2}\omega_{aa,\gamma}}\right)$ [A11]

where terms are as defined in equation 10. Allele frequency changes in the free-living life stages are independent of the sheep genotype (*γ*).

**Changes to worm allele frequencies when transitioning from parasitic to free-living life stages**

The reproduction parameter (*W_F,γ_*) means that the allele frequency of eggs may be different to the allele frequency in the parental worm population. Hence, the allele frequency for eggs produced in age-class *i* reflects the relative reproductive success of each particular genotype. It is given by:

$p_{i,j,t} = \frac{1}{2}\left\{\frac{p_{i,j,t}^{2}\omega_{AA,\gamma}+p_{i,j,t} q_{i,j,t}\omega_{Aa,\gamma}}{p_{i,j,t}^{2}\omega_{AA,\gamma}+2p_{i,j,t}q_{i,j,t}\omega_{Aa,\gamma}+ q_{i,j,t}^{2}\omega_{aa,\gamma}}\right\} + \frac{1}{2}\sum_{i=21}^{I} \left\{\frac{B_{i,j,t}}{\sum B_{21:I,j,t}}p_{i,j,t}\right\}$ i=21..I

[A12]

where terms are as defined in equation 10. The first part of the equation describes the genotypes of female parents, i.e. female adult worms of age-class *i*, while the second part reflects the genotypes of their males mates, assuming that females mate randomly (or with equal probability) with male worms from all age groups within an individual sheep. This approximates the polyandrous behaviour observed in some trichostrongylid species . Allele frequency for eggs on pasture is then the allele frequency from each age-class (equation A12), weighted by each hosts age-class contribution to new eggs on pasture (*C_1,t_*, equation A5). Hence,

$p_{1,t} = \sum_{j=1}^{J} \sum_{i=1}^{I} \left\{\frac{p_{i,j,t} O_{i,j,t}}{C_{1,t}}\right\}$ [A13]

**Changes to worm allele frequency from free-living to parasitic life stages**

The genotype for ingested worms is the sum of allele frequencies for infective larvae, weighted by their relative contribution to total infective larvae on pasture (i.e. *∑C_7:I,t_*).

$p_{j,t+1} = \frac{\sum_{i=7}^{I} p_{i,j,t} C_{i,t}}{\sum C_{7:I,t}}$ [A14]

The allele frequency for new worms in sheep *j* (*B_1,j,t_,* equation A9) is then calculated using the average allele frequency from equation A14, and the appropriate host establishment parameters for the worm and host genotypes (i.e. *ω_AA,γ_, ω_Aa,γ_, ω_aa,γ_*).

**Development of host immunity**

Sheep phenotypes for worm establishment, adult worm mortality and female worm fecundity are changed daily to approximate the development of immunity with increasing larval exposure, reflecting observations made under field conditions. Phenotypes are changed according to:

$E_{j,t}=E_{j,0} e^{-q t}$ [A15]

$M_{j,t}=\frac{M_{j,0} t^{2}}{m+t^{2}}$ [A16]

$F_{j,t}=\left\{\frac{F_{j,0}f^{2}}{f^{2}+t^{2}}\right\}F_{den}$ [A17]

, where the constants *q*, *m*, and *f* control the shape of the curves; *E_j,0_, M_j,0_* and *F_j,0_* are the initial phenotypes for sheep *j* at time 0 and *F_den_* is the density-dependent function for worm fecundity. Density-dependent effects for female fecundity were assumed to occur if the total adult worm burden for an individual exceeded a threshold *β* (i.e. if *∑B_21:I,j,t_* > *β*), otherwise *F_den_* = 1. If *∑B_21:I,j,t_* > *β* then

$F_{den}=\exp\left(\frac{\beta}{\sum B_{21 :I,j,t}}-1\right)$ [A18]

This equation reduces adult fecundity as a function of the worm burden, as seen under field conditions . Examples of these time-dependent changes to phenotypes and density dependent effects are shown in Figure A2.

| ***A.*** | ***B.*** |
| --- | --- |
| ***C.*** | ***D.*** |

Figure A2 Increased immunity leads to reduced worm establishment (A), increased adult mortality (B) and reduced female worm fecundity (C) phenotypes for sheep over time. Density dependent constraints may also restrict female worm fecundity (D). Shown are mean (solid) and extreme (broken lines, ± 2 standard deviations) sheep (polygenic) phenotypes

Calculating faecal worm egg count (WEC). WEC is calculated at day 100 as

[A19]

where *δ* is the indigestibility of the diet (1-digestability, g/kg). WEC is not used as input in the worm infection or animal modules.

## Parameterising the model

Parameter values were chosen to reflect our previously published data . That is, for an intake of about 2500 larvae per animal, the model was parameterised to achieve a mean WEC of 720 epg and adult burden of 18 000 worms. Defining this unselected population was challenging because of the dynamic nature of the model, including daily changes to the sheep phenotypes and between-animal variation. Parameters were explored starting with population means for daily mortality of 0.06 and a daily mortality rate for 3^rd^ stage larvae on pasture (*ρ*) of 0.05. The phenotypic variance in each trait was assumed to be similar to Kao *et al.* . Parameters were adjusted until the desired larval intake, adult worm burden and WEC were achieved. Final values for constants and the polygenic components for sheep phenotypes are as shown Tables A2 and A3.

Table A3. Phenotypic and genetic components for worm establishment (*E_j,0_*), adult worm mortality (*M_j,0_*) and female worm fecundity (*F_j,0_*) for sheep. Shown is the polygenic mean (*μ_poly_*) and variance (*σ^2^_poly_*), phenotypic variance (*σ^2^_P_*), co-efficient of variation (CV) and polygenic heritability (*h^2^_poly_*)

|  | | Genetic component | | Phenotype | |
| --- | --- | --- | --- | --- | --- |
|  | *μ_poly_* | *σ^2^_poly_* | *σ^2^_P_* | CV | *h^2^_poly_* |
| E^1^ | -1.0 | 4.8x10^-2^ | 0.16 | 0.40 | 0.30 |
| M | 0.080 | 1.2x10^-4^ | 4.0x10^-4^ | 0.25 | 0.30 |
| F | 70 | 240 | 800 | 0.40 | 0.30 |

^1^genetic component described prior to transformation, transformed mean 0.37

## References

1. Falconer DS, Mackay TFC: *Introduction to Quantitative Genetics.* 4th ed. edn. Edinburgh, UK: Pearson Education Limited; 1996.

2. Redman E, Grillo V, Packard E, Saunders G, Jackson F, Berriman M, Gilleard J: **Genetics of mating and sex determination in the parasitic nematode *Haemonchus contortus*.** *Genetics* 2008, **180:**1877-1887.

3. Louie K, Vlassoff A, Mackay A: **Nematode parasites of sheep: Extension of a simple model to include host variability.** *Parasitology* 2005, **130:**437-446.

4. Bishop SC, Stear MJ: **The use of a gamma-type function to assess the relationship between the number of adult *Teladorsagia circumcincta* and the total egg output.** *Parasitology* 2000, **121:**435-440.

5. Kemper KE, Palmer DG, Liu SM, Greeff JC, Bishop SC, Karlsson LJE: **Reduction of faecal worm egg count, worm numbers and worm fecundity in sheep selected for worm resistance following artificial infection with *Teladorsagia circumcincta* and *Trichostrongylus colubriformis*.** *Veterinary Parasitology* 2010, **171:**238-246.

6. Bishop SC, Stear MJ: **Modelling responses to selection for resistance to gastrointestinal parasites in sheep.** *Animal Science* 1997, **64:**469-478.

7. Kao RR, Leathwick DM, Roberts MG, Sutherland IA: **Nematode parasites of sheep: A survey of epidemiological parameters and their application in a simple model.** *Parasitology* 2000, **121:**85-103.
